# Indoxyl Sulfate Promotes Arterial Thrombosis in Rat Model via Increased Levels of Complex TF/VII, PAI-1, Platelet Activation as Well as Decreased Contents of SIRT1 and SIRT3

**DOI:** 10.3389/fphys.2018.01623

**Published:** 2018-11-28

**Authors:** Malgorzata Karbowska, Tomasz W. Kaminski, Beata Znorko, Tomasz Domaniewski, Tomasz Misztal, Tomasz Rusak, Anna Pryczynicz, Katarzyna Guzinska-Ustymowicz, Krystyna Pawlak, Dariusz Pawlak

**Affiliations:** ^1^Department of Pharmacodynamics, Medical University of Bialystok, Bialystok, Poland; ^2^Department of Monitored Pharmacotherapy, Medical University of Bialystok, Bialystok, Poland; ^3^Department of Physical Chemistry, Medical University of Bialystok, Bialystok, Poland; ^4^Department of General Pathomorphology, Medical University of Bialystok, Bialystok, Poland

**Keywords:** indoxyl sulfate, arterial thrombosis, chronic kidney disease, tissue factor, SIRT1, SIRT3, hemostatic disorder, sirtuin

## Abstract

Patients suffering from chronic kidney disease (CKD) are at a 20-fold higher risk of dying due to cardiovascular diseases (CVDs), primarily thrombosis following vascular injury. CKD is connected with retention of uremic toxins, especially indoxyl sulfate (IS), which are currently considered as a non-classical CKD-specific risk factor for CVDs. The present study aimed to examine the effect of chronic exposure to IS on the hemostatic system and arterial thrombosis in a model without greater interferences from the uremic milieu consisting of additional uremic toxins. Forty-eight male Wistar Crl:WI (cmdb) rats were divided into three groups: one control group and two experimental groups, which were exposed to 100 or 200 mg/kg of b.w./day of IS in drinking water for a period of 28 days. The control group received water without IS. At the end of the experiment, the induction of arterial thrombosis was performed. We investigated the impact of IS on thrombosis incidence, kinetics and strength of clot formation, platelet activity, aortic contents of sirtuin (SIRT) 1 and sirtuin 3 (SIRT3), hemostatic system, cardiorespiratory parameters, biochemistry of plasma and urine as well as histology of the thrombus, kidney, and liver. Obtained data revealed that chronic exposure to IS promotes arterial thrombosis via increased levels of complex tissue factor/factor VII, plasminogen activator inhibitor-1 (PAI-1), platelet activation, as well as decreased aortic levels of SIRT1 and SIRT3. Therefore, we hypothesize that IS enhances primary hemostasis leading to augmented formation of platelet plug with increased amounts of fibrin and affects secondary hemostasis through the influence on plasma coagulation and fibrinolysis factors, which results in the increased kinetics and strength of clot formation. The findings described may contribute to a better understanding of the mechanisms leading to increased thrombotic events in patients with CKD with elevated levels of IS.

## Introduction

Chronic kidney disease is a growing global health problem that affects almost 15% of the world population (Eknoyan et al., 2004). Patients suffering from CKD are at a 20-fold higher risk of dying due to CVDs, especially due to thrombosis following vascular injury. CKD is accompanied by about 10-fold increased risks of thrombotic events (Burmeister et al., 2014). Despite improved diagnostic capabilities, the etiopathogenesis of thrombotic events in CKD is still not fully understood.

Chronic kidney disease is associated with the retention of uremic toxins that are currently considered as a non-classical CKD-specific risk factor for CVD (Fujii et al., 2018). One of the most potent uremic toxins is IS, whose concentration can be increased almost by 80 times during renal insufficiency (Karbowska et al., 2016; Hung et al., 2017). Our previous work revealed the prothrombotic properties of acutely administrated IS, which were observed by the acceleration of thrombotic response following vascular injury in mouse and rat models (Karbowska et al., 2017). Moreover, other studies have provided evidence that the prothrombotic effect of IS is associated with the activation of TF via stimulation of AhRs (Chitalia et al., 2013; Shivanna et al., 2016). Shashar et al. (2017) reported that IS augments thrombosis after vascular injury through the reduction in STUB1–TF interaction in vascular smooth muscle cells. In turn, Yang et al. (2017) suggest that IS contributes to platelet hyperactivity. Even though IS is included in a group termed as “thrombolome” (Shashar et al., 2015) and considered as a link between hemostatic disturbances and CVD prevalence (Kaminski et al., 2017), there are no data showing the influence of IS on particular parameters of coagulation and fibrinolysis in a model without the interferences of the uremic milieu, consisting of additional uremic toxins.

Sirtuins – Sir2 proteins – are members of the class III type nicotinamide adenine dinucleotide-dependent histone deacetylases. Their roles are commonly linked to aging, metabolism, and antioxidant defense (Winnik et al., 2015). Furthermore, some of the sirtuins, such as Sirtuin 1, exert renal protective effects by maintaining the function of the glomerular barrier, participating in the regulation of blood pressure and sodium balance, and inhibiting renal cell apoptosis and fibrosis. Additionally, Sirtuin 3 has antioxidant and anti-inflammatory properties that can result in the reduction of lipotoxicity-induced inflammation and protect renal tubular cells (Kitada et al., 2013; Guan and Hao, 2015; Wakino et al., 2015). In addition to the mentioned roles of sirtuins, recent studies report that the inhibition of some sirtuins may exert prothrombotic effects. Breitenstein et al. (2011) showed that the inhibition of Sirt1 contributes to arterial thrombosis associated with the increased activation of nuclear factor-kappa B/p65, leading to augmented TF activity. Similarly, Gaul et al. (2018) noticed that genetic loss of Sirtuin 3 results not only in enhanced TF activity, but also increases the formation of NETs that together lead to the promotion of arterial thrombosis. Little is known about the impact of IS on sirtuins. Koizumi et al. (2014) demonstrated that IS decreases Sirt1 activity through the activation of AhR in an *in vitro* model. However, there are no literature data currently available regarding the influence of IS on Sirt1 and Sirt3 and its involvement in CKD thrombosis in *in vivo* studies.

The present study is a continuation of our previous work (Karbowska et al., 2017) and it aimed to examine the effect of chronic exposure to IS on the hemostatic system and arterial thrombosis in a rat model focusing on observing the effect of IS only. We confirmed that IS-induced arterial thrombosis after chronic administration increased the kinetics and strength of clot formation, and contributed to platelet hyperactivity as well as reduced aortic levels of SIRT1 and SIRT3.

## Materials and Methods

### Animals

Male Wistar rats obtained from the Center of Experimental Medicine in the Medical University of Bialystok were housed in a temperature- and humidity-controlled room according to Good Laboratory Practice rules. Rats were allowed to have *ad libitum* access to sterilized tap water and standard chow in specific pathogen-free conditions. The number of animals was estimated after the calculation of the sample size. All procedures involving animals were approved by a Local Ethics Committee (Agreement Nos. 124/2015 and 31/2017/WNP) and conducted in accordance with institutional guidelines and the principles of the Basel Declaration.

### Experimental Design

Forty-eight male Wistar Crl:WI (cmdb) rats weighing 180–210 g were divided into three groups: one control group (CON, *n* = 16) and two experimental groups, which were exposed to 100 mg/kg of b.w./day (100 IS, *n* = 16) or 200 mg/kg of b.w./day (200 IS, *n* = 16) of IS (IS was used in the form of potassium salt provided by Sigma-Aldrich) in drinking water for a period of 28 days (Table 1). The control group received pure water without IS. At the end of each experiment, rats were placed in metabolic cages for 24-h urine collection. The urine was aliquoted and frozen at -80°C until assays were performed. Next, rats were weighed and anesthetized with pentobarbital (40 mg/kg b.w., i.p.), and the induction of arterial thrombosis was performed. Subsequently, blood samples were taken from the left ventricle and centrifuged to obtain plasma. After centrifugation, plasma was stored and frozen at -80°C until assays were performed. The kidneys, livers, and thrombi were also removed and weighed, and representative samples were fixed in 10% neutral formalin.

**Table 1:** Differences between acute and chronic exposure to indoxyl sulfate in animal model.

	Acute exposure (Karbowska et al., 2017)	Chronic exposure
**Main differences in methodology**		
Model	Rats and mice	Rats
Duration of exposure to IS	Single injection (*in vivo*, *ex vivo* tests) or 30–45 min incubation (*in vitro* tests)	28 days
Route of IS administration	Intravenous	Dissolved in the drinking water
Solvent	0.9% NaCl	Water
IS doses	3, 10, 30, and 100 mg/kg of b.w. in rats; 30 and 100 mg/kg of b.w. in mice	100 or 200 mg/kg of b.w.
Model of thrombosis	Arterial thrombosis induced by direct electric current in rats and thrombosis after laser-induced injury in mice	Arterial thrombosis induced by direct electric current in rats
**Main differences in the obtained results of the same kind of tests**		
IS plasma concentration assessed by HPLC	From ∼0.1 to 1.7 mM (Karbowska et al., 2017)	From ∼20 to 200 uM
ROTEM analysis	Increase in AUC and decrease in CT (Karbowska et al., 2017)	No changes in AUC and CT
Coagulation parameters	Decrease in APTT and fibrinogen (Karbowska et al., 2017)	No changes in APTT and increase in fibrinogen
Blood morphology parameters	No changes in MCH, MCV, PLT, RBC, and WBC (Karbowska et al., 2017)	Increase in MCH, MCV, PLT and WBC and decrease in RBC

*AUC, area under the curve; APTT, activated partial thromboplastin time; b.w., body weight; CT, clotting time; IS, indoxyl sulfate; MCH, mean corpuscular hemoglobin; MCV, mean corpuscular volume; PLT, platelet; RBCs, red blood cells; WBCs, white blood cells*.

### Determination of IS

Concentrations of the free form of IS in the plasma and urine were evaluated using HPLC with fluorescence detection according to the modifications (Karbowska et al., 2017) we made in the methods described previously by Al Za’abi et al. (2013).

### Experimental Arterial Thrombosis in Rats

Arterial thrombosis was induced by electrical stimulation of the right common carotid artery as has been previously described in detail (Karbowska et al., 2017). After 60 min of the stimulation, the thrombus formed was completely removed, air-dried at 37°C (24 h), and weighed.

### Analysis of Hemostatic Parameters

The following hemostatic parameters were determined in the rats’ plasma by ELISA kits (Cloud-Clone, Corp., Houston, TX, United States): PF4, vWF, FV, FXa, TF, TF/VII complex, TAFI, PAI-1, PAP, and d-dimers.

### Evaluation of Coagulation Profile

Activated partial thromboplastin time, PT, TT, concentration of fibrinogen, and AT III activity were measured in citrated plasma using coagulometer Coag-Chrom 3003 (BioKsel, Grudziadz, Poland) and Bio-Ksel plasma kits.

### ROTEM Analysis

The evaluation of the effect of chronic exposure to IS on the dynamics of clot formation was performed using ROTEM technology as described previously (Misztal et al., 2015; Karbowska et al., 2017). Analysis was performed using the ROTEM system (Tem International GmbH, Mannheim, Germany). Coagulation was triggered by the addition of calcium chloride (12 mM final concentration) to the samples and the measurements were performed at least 35 min to the moment when the following parameters were collected: alpha angle (°) — the angle showing the dynamics of clot formation, AUC — indicating the maximum clot formation, CT — time from start of measurement to the beginning of the fibrin polymerization process, MCF — reflecting the strength of the formed clot to resist the pin oscillation, and CFT — time from start of measurement to stable clot formation.

### Analysis of Morphological Parameters

The following blood morphology parameters were evaluated in whole blood samples using Animal Blood Counter (ABC Vet, Horiba, Viernheim, Germany) according to the manufacturer’s directions: WBCs, RBCs, HGB, HCT, MCV, MCH, MCHC, and PLTs.

### Plasma and Urine Biochemistry

Plasma biochemical parameters such as ALAT, ASAT, total cholesterol, TGs, LDL-d, HDL, creatinine, UA, urea, and total proteins, as well as urinary biochemical parameters such as creatinine and urine proteins were measured using an automated biochemical analyzer (Mindray BS-120, Mindray, Mahwah, NJ, United States).

### Histological Evaluation of Thrombi, Kidneys, and Livers

Collected tissues were fixed in formalin and processed routinely for embedding in paraffin. Sections of thrombi, kidneys, and livers were cut on a microtome at a thickness of 4 μm and stained by H&E and additionally by PAS in the case of kidneys. The analysis of renal microscopical findings was performed using the semiquantitative method for the following histopathological indicators of tubular injury: interstitial fibrosis, tubular atrophy, and interstitial inflammation (Cao et al., 2000). Additionally, PAS staining was made in the case of kidneys to highlight the basement membranes of tissues and analyze the degree of renal glomerulosclerosis. All histological evaluations of thrombi, kidneys, and livers were performed randomly under the light microscope (Olympus CX40). Tissues were assessed independently by two observers.

### Monitoring of the Cardiorespiratory Parameters

The cardiorespiratory parameters: heart rate, tissue perfusion of rat’s paw, and SpO_2_ were monitored during 50 min after electrical stimulation of the common carotid artery using a rodent vital sign monitor (Physiological Monitor, PhysioSuite, Kent Scientific Corporation, United States). Body temperature was monitored via a rectal probe (PhysioSuite, Kent Scientific Corporation, United States).

### Measurement of SIRT1 and SIRT3 Levels in Aortas

Aortic levels of SIRT1 and SIRT3 in homogenates (1:25) were measured using rat ELISA kits supplied by Cloud-Clone, Corp., Houston, TX, United States according to the manufacturer’s instructions.

### Statistical Analysis

Shapiro–Wilk’s test of normality was used for data distribution analysis. The normally distributed data were presented as mean ± SD, while the non-Gaussian data were presented as median (full-range). Comparison between data was made by Student’s *t*-test or non-parametric Mann–Whitney *U*-test. The correlations were calculated by Spearman’s rank correlation analysis. A two-tailed *p*-value < 0.05 was considered statistically significant. Statistical analyses were performed with GraphPad 6 Prism (GraphPad Software, La Jolla, CA, United States).

## Results

### General Characteristics of Rats

The general characteristics of rats are shown in Table 2. There were no differences in the fluid and food intake, 24-h urine volume, final body weight, and weight of all mentioned organs except kidneys. The kidneys’ weight was higher in the 200 IS group (*p* < 0.05). The 100 IS and 200 IS groups presented higher plasma concentrations of ALAT, total cholesterol, HDL, and creatinine (*p* < 0.05). Only the 200 IS group had increased concentration of UA in plasma (*p* < 0.05) and urine protein excretion (*p* < 0.01). Creatinine clearance was decreased in both experimental groups (*p* < 0.05).

**Table 2:** General characteristics of rats.

	CON	100 IS	200 IS
Final body weight [g]	345.5 ± 17.6	348.0 ± 17.3 NS	350.8 ± 24.8 NS
Fluid intake [ml/day]	37.69 ± 4.9	35.76 ± 1.5 NS	35.76 ± 2.9 NS
Food intake [g/day]	28.93 ± 2.9	30.62 ± 1.7 NS	30.29 ± 3.8 NS
24-h urine volume [ml]	23.75 ± 7.7	19.75 ± 7.8 NS	20.06 ± 7.7 NS
Liver weight [g]	8.99 (7.98–11.83)	9.39 (8.00–12.17) NS	9.27 (7.99–14.99) NS
Liver/body weight ratio	0.0263 (0.0240–0.0355)	0.0263 (0.0236–0.0364) NS	0.0268 (0.0241–0.0405) NS
Kidneys weight [g]	1.24 (0.92–1.53)	1.29 (0.90–1.65) NS	1.33 (1.15–1.68)^∗^
Kidneys/body weight ratio	0.0036 ± 0.0003	0.0037 ± 0.0004 NS	0.0038 ± 0.0003 NS
ASAT [U/L]	141.5 (84–198)	131.0 (102–281) NS	154.5 (97–326) NS
ALAT [U/L]	34.0 (25–82)	41.0 (34–105)^∗^	44.0 (32–124)^∗^
Cholesterol total [mg/dL]	35.42 ± 6.2	41.45 ± 7.4^∗^	39.77 ± 2.5^∗^
TG [mg/dL]	43.15 ± 10.8	40.92 ± 4.9 NS	47.55 ± 16.7 NS
LDL-d [mg/dL]	2.34 ± 0.9	3.23 ± 1.0 NS	2.57 ± 0.7 NS
HDL-d [mg/dL]	15.98 ± 2.4	18.37 ± 2.1^∗^	18.08 ± 2.1^∗^
Creatinine in plasma [mg/dL)	0.28 ± 0.08	0.36 ± 0.07^∗^	0.39 ± 0.14^∗^
UA [mg/dL]	0.57 ± 0.3	0.63 ± 0.2 NS	0.88 ± 0.4^∗^
Urea [mg/dL]	49.92 ± 6.7	49.00 ± 7.0 NS	53.23 ± 7.7 NS
BUN [mg/dL]	22.96 ± 3.2	23.04 ± 2.9 NS	24.49 ± 3.5 NS
Total proteins [g/dL]	5.58 (5.35–6.23)	5.62 (5.32–6.37) NS	5.67 (5.40–5.92) NS
Creatinine in urine [mg/dL]	40.20 (22.99–80.47)	47.24 (21.81–85.14) NS	48.20 (29.11–80.43) NS
Urine proteins excretion [mg/24 h]	10.11 ± 1.3	11.61 ± 3.0 NS	12.82 ± 2.8^∗∗^
Urine proteins/creatinine ratio	1.13 ± 0.18	1.25 ± 0.35 NS	1.45 ± 0.46^∗^
Creatinine clearance [ml/min]	2.38 ± 0.8	1.69 ± 0.5^∗^	1.65 ± 0.7^∗^

*Data are shown as the mean ± SD or median (full range) depending on their distribution.*

*CON, control group; 100 IS, group receiving IS in the dose of 100 mg/kg b.w./day; 200 IS, group receiving IS in the dose of 200 mg/kg b.w./day; ALAT, alanine aminotransferase; ASAT, aspartate aminotransferase; BUN, blood urea nitrogen; HDL-d, high-density lipoprotein direct; IS, indoxyl sulfate; LDL-d, low-density lipoprotein direct; NS, non-significant; TG, triglycerides; UA, uric acid; ^∗^*p* < 0.05 compared to control; ^∗∗^*p* < 0.01 compared to control*.

### Concentrations of IS in the Rats’ Plasma and Urine

**FIGURE 1:**
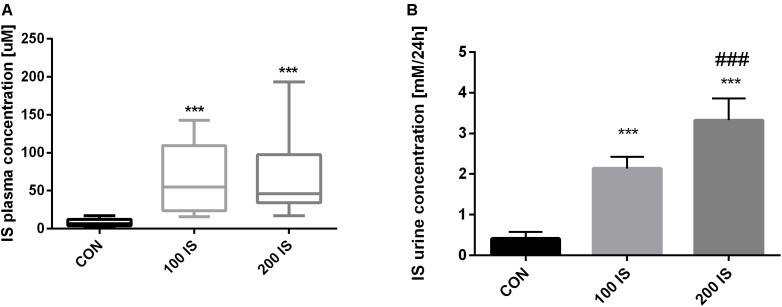
The plasma **(A)** and urine **(B)** concentrations of IS after chronic administration. Data are presented as the mean ± SD or median (full range) depending on their distribution. CON, control group; 100 IS, group receiving IS in the dose of 100 mg/kg b.w./day; 200 IS, group receiving IS in the dose of 200 mg/kg b.w./day; IS, indoxyl sulfate; ^∗∗∗^*p* < 0.001 compared to control; ^###^*p* < 0.001 compared to 100 IS group.

Chronic exposure to IS in the doses of 100 and 200 mg/kg b.w. led to statistically significant increases in IS concentrations when compared to the control group (*p* < 0.001) (Figure 1A). Similarly, concentrations of IS in the urine collected during 24-h were higher in both experimental groups (*p* < 0.001). However, the concentration of IS in the urine in the 200 IS group was also increased when compared to the 100 IS group (*p* < 0.001) (Figure 1B).

### Effect of IS on Formation of Thrombus

**FIGURE 2:**
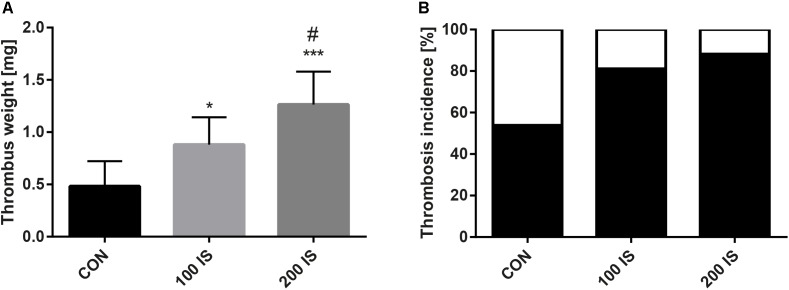
**(A)** The weight of developed thrombus in arterial thrombosis model. **(B)** The frequency of incidence of thrombosis. Data from **(A)** are shown as the mean ± SD. CON, control group; 100 IS, group receiving IS in the dose of 100 mg/kg b.w./day; 200 IS, group receiving IS in the dose of 200 mg/kg b.w./day; IS, indoxyl sulfate; ^∗^*p* < 0.05 compared to control; ^∗∗∗^*p* < 0.001 compared to control; ^#^*p* < 0.05 compared to 100 IS group.

Rats chronically exposed to IS were characterized by significantly increased thrombus weight when compared to the control group (*p* < 0.05, 100 IS; *p* < 0.001, 200 IS) (Figure 2A). Moreover, the highest dose of IS augmented the thrombus weight when compared with the dose of 100 mg/kg b.w. (*p* < 0.05). In addition, the frequency of thrombosis incidence was also higher in both experimental groups than in the control group (Figure 2B).

### Impact of IS on the Kinetics and Strength of Clot Formation

**FIGURE 3:**
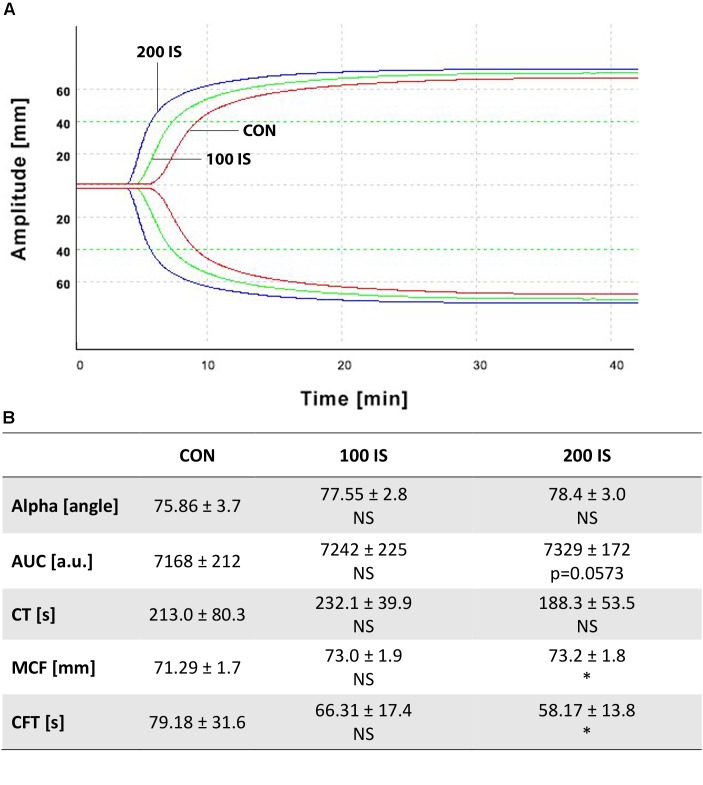
**(A)** Graphical representation of influence of IS on ROTEM analyses. **(B)** Impact of IS on kinetics and strength of clot formation. Data from **(B)** are shown as the mean ± SD. CON, control group; 100 IS, group receiving IS in the dose of 100 mg/kg b.w./day; 200 IS, group receiving IS in the dose of 200 mg/kg b.w./day; a.u., arbitrary units; AUC, area under the curve; CFT, clot formation time; CT, clotting time; IS, indoxyl sulfate; MCF, maximum clot firmness; NS, non-significant; ^∗^*p* < 0.05 compared to control.

As shown in Figure 3, only the highest IS dose increased MCF (*p* < 0.05) and decreased CFT (*p* < 0.05) when compared with control. Other parameters were not affected. Furthermore, we did not observe any changes in the 100 IS group.

### Influence of IS on Coagulation Parameters

Chronically administered IS did not influence parameters like APTT, PT, TT, and activity of ATIII. Only the IS dose of 200 mg/kg b.w. led to an increase in the concentration of fibrinogen (*p* < 0.05) (Table 3).

**Table 3:** Influence of IS on coagulation parameters.

	CON	100 IS	200 IS
APTT [s]	20.01 ± 1.8	19.8 ± 3.8 NS	18.2 ± 2.9 NS
Fibrinogen [g/L]	2.29 (1.95–2.45)	2.40 (1.95–2.45) NS	2.40 (2.32–2.50) ^∗^
PT [s]	20.9 (12.9–23.1)	21.4 (12.4–28.3) NS	19.5 (12.1–26.2) NS
TT [s]	25.90 ± 4.2	24.80 ± 3.9 NS	26.70 ± 3.6 NS
AT III [%]	86.5 (75.2–101)	87.3 (62.4–103) NS	86.3 (70.7–96.3) NS

*Data are shown as the mean ± SD or median (full range) depending on their distribution.*

*CON, control group; 100 IS, group receiving IS in the dose of 100 mg/kg b.w./day; 200 IS, group receiving IS in the dose of 200 mg/kg b.w./day; APTT, activated partial thromboplastin time; AT III, antithrombin III; IS, indoxyl sulfate; NS, non-significant; PT, prothrombin time; TT, thrombin time; ^∗^*p* < 0.05 compared with control*.

### IS-Induced Changes in Hemostatic Parameters

**FIGURE 4:**
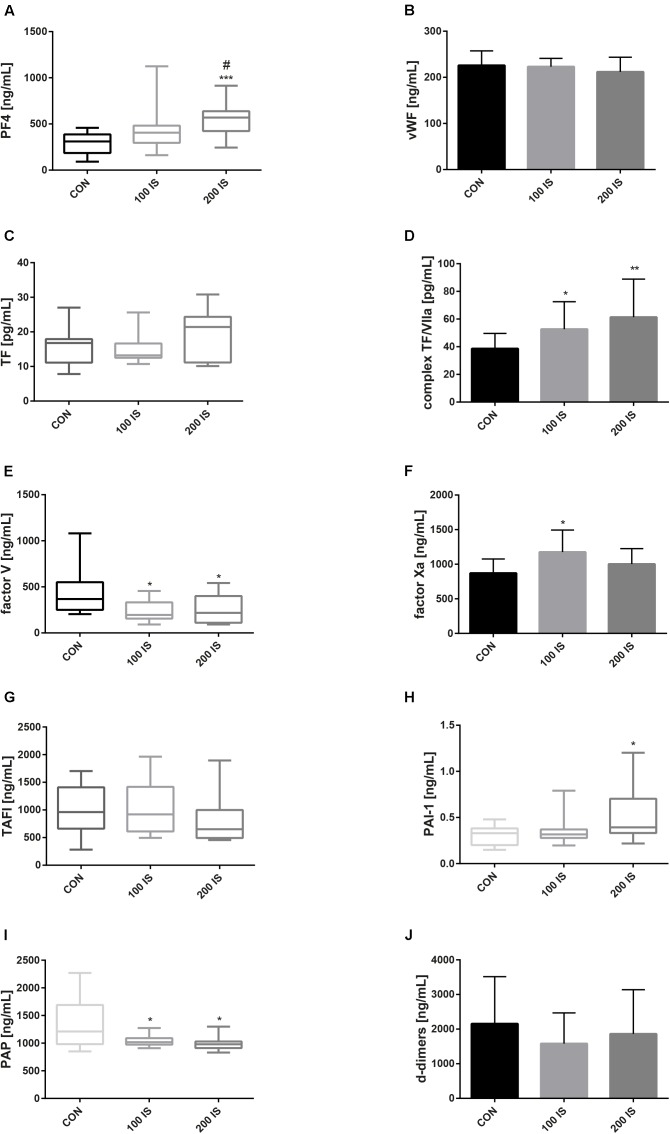
IS-induced changes in hemostatic parameters: PF4 **(A)**, vWF **(B)**, TF **(C)**, TF/VII complex **(D)**, FV **(E)**, FXa **(F)**, TAFI **(G)**, PAI-1 **(H)**, PAP **(I)**, and d-dimers **(J)**. Data are presented as the mean ± SD or median (full range) depending on their distribution. CON, control group; 100 IS, group receiving IS in the dose of 100 mg/kg b.w./day; 200 IS, group receiving IS in the dose of 200 mg/kg b.w./day; IS, indoxyl sulfate; PF4, platelet factor 4; vWF, von Willebrand factor; FV, coagulation factor V; FXa, activated coagulation factor X; TF, tissue factor; TF/VII complex, TF/coagulation factor VII complex; TAFI, thrombin activatable fibrinolysis inhibitor; PAI-1, plasminogen activator inhibitor-1; PAP, plasmin-alpha2-antiplasmin complex; ^∗^*p* < 0.05 compared to control; ^∗∗^*p* < 0.01 compared to control; ^∗∗∗^*p* < 0.001 compared to control; ^#^*p* < 0.05 compared to 100 IS group.

The 100 IS and 200 IS groups presented a lower level of FV and PAP when compared to the control group (*p* < 0.05). In turn, concentrations of TF/VII complexes were significantly higher in the IS groups (*p* < 0.05, 100 IS; *p* < 0.01, 200 IS, compared to the control group). Moreover, the 200 IS group showed augmented concentrations of PAI-1 (*p* < 0.05 compared to the control group) and PF4 – a marker of platelet activation of which clearance is not dependent on the functioning kidney tissue (Bastl et al., 1981). A statistically significant increase in FXa concentration compared to control was observed only in the 100 IS group (*p* < 0.05). There were no differences in concentrations of vWF, TF, TAFI, and d-dimers (Figure 4).

### Effect of IS on Levels of SIRT1 and SIRT3 in Aortas

**FIGURE 5:**
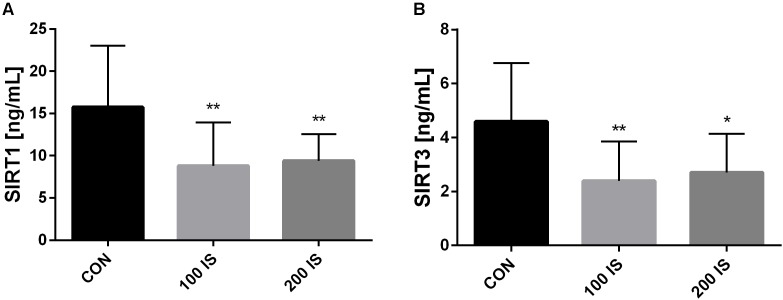
The effect of IS on SIRT1 **(A)** and SIRT3 **(B)** levels in aortas. Data are presented as the mean ± SD. CON, control group; 100 IS, group receiving IS in the dose of 100 mg/kg b.w./day; 200 IS, group receiving IS in the dose of 200 mg/kg b.w./day; IS, indoxyl sulfate; SIRT1, sirtuin 1; SIRT3, sirtuin 3; ^∗^*p* < 0.05 compared to control; ^∗∗^*p* < 0.01 compared to control.

The ELISA kits revealed that chronic exposure to IS reduced aortic contents of SIRT1 (*p* < 0.01 both doses) as well as SIRT3 (*p* < 0.01, 100 IS; *p* < 0.05, 200 IS) when compared to control group (Figure 5).

### Blood Morphology Parameters

Chronic administration of IS in the doses of 100 and 200 mg/kg b.w. resulted in increased PLT (*p* < 0.05). Additionally, the 200 IS group presented higher WBC when compared to control (*p* < 0.05). Moreover, the highest dose of IS decreased RBC (*p* < 0.001 compared to control; *p* < 0.01 compared to 100 IS) whereas it increased MCH and MCV (both *p* < 0.001 compared to control and 100 IS). The HCT, HGB, and MCHC did not differ between all studied groups (Table 4).

**Table 4:** Effect of IS on blood morphology parameters.

	CON	100 IS	200 IS
HCT [%]	47.0 (35.4–49.7)	48.3 (45.3–56.1) NS	47.25 (43.1–53.9) NS
HGB [g/dl]	15.94 ± 0.6	15.94 ± 0.7 NS	15.74 ± 0.9 NS
MCH [pg]	20.12 ± 0.7	20.61 ± 0.5 NS	21.66 ± 0.8^∗∗∗^ ###
MCHC [g/dl]	35.5 (32.7–37.3)	33.6 (33.4–34.2) NS	33.4 (32.7–34.5) NS
MCV [um^3^]	59.36 ± 1.4	61.07 ± 1.5 NS	64.33 ± 2.6^∗∗∗^ ###
PLT [10 × 3/mm^3^]	490.5 ± 31.65	528.4 ± 48.95^∗^	533.4 ± 44.91^∗^
RBC [10 × 6/mm^3^]	7.93 ± 0.4	7.76 ± 0.3 NS	7.27 ± 0.5^∗∗∗^ ##
WBC [10 × 3/mm^3^]	2.6 (1.5–9.5)	3.3 (2.2–8.5) NS	3.4 (2.2–14.0)^∗^

*Data are shown as the mean ± SD or median (full range) depending on their distribution.*

*CON, control group; 100 IS, group receiving IS in the dose of 100 mg/kg b.w./day; 200 IS, group receiving IS in the dose of 200 mg/kg b.w./day; HCT, hematocrit; HGB, hemoglobin; IS, indoxyl sulfate; MCH, mean corpuscular hemoglobin; MCHC, mean corpuscular hemoglobin concentration; MCV, mean corpuscular volume; NS, non-significant; PLT, platelet; RBCs, red blood cells; WBCs, white blood cells; ^∗^*p* < 0.05 compared to control; ^∗∗∗^*p* < 0.001 compared to control; ^##^*p* < 0.01 compared to 100 IS group; ^###^*p* < 0.001 compared to 100 IS group*.

### Histology of Thrombus, Kidney, and Liver

**FIGURE 6:**
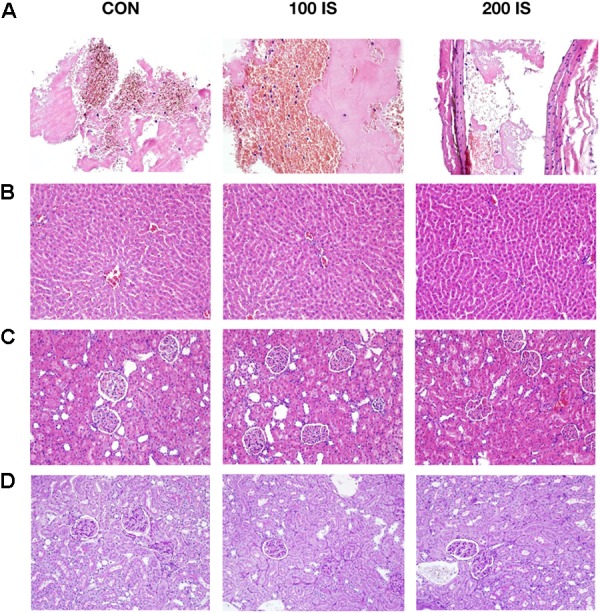
Representative examples of the thrombi **(A)**, livers **(B)**, and kidneys **(C,D)** histology. Samples **(A–C)** were stained by hematoxylin and eosin (H&E). Samples **(D)** were stained by periodic acid schiff (PAS). Images **(A)** were taken using 400× magnification. Images **(B–D)** were taken using 200× magnification. CON, control group; 100 IS, group receiving IS in the dose of 100 mg/kg b.w./day; 200 IS, group receiving IS in the dose of 200 mg/kg b.w./day; IS, indoxyl sulfate.

Figure 6 shows representative examples of histology of thrombi (Figure 6A), livers (Figure 6B), and kidneys (Figures 6C,D). Histological examination revealed that thrombi of IS-exposed rats were richer in fibrin with PLT (about 70% of fibrin with PLT in relation to RBC) when compared to control rats (about 50% of fibrin with PLT in relation to RBC). The kidneys and the liver of the control and experimental groups were normal under an optical microscope without evident alterations of the structure.

### Cardiorespiratory Parameters

The IS did not alter heart rate, tissue perfusion, and SpO_2_. Similar values of body temperature were observed in all the groups (Table 5).

**Table 5:** Impact of IS on cardiorespiratory parameters.

	CON	100 IS	200 IS
Heart rate [bpm]	396.5 (363–418)	391 (372–452) NS	412 (364–453) NS
Perfusion [%]	0.0675 (0.044–0.118)	0.0547 (0.029–0.146) NS	0.0401 (0.025–0.138) NS
SpO_2_ [%]	65.89 ± 10.7	66.94 ± 9.9 NS	67.78 ± 5.6 NS
Body temperature [°C]	36.4 (35.1–36.8)	36.5 (36.0–37.7) NS	36.6 (36.3–38.2) NS

*Data are shown as the mean ± SD or median (full range) depending on their distribution.*

*CON, control group; 100 IS, group receiving IS in the dose of 100 mg/kg b.w./day; 200 IS, group receiving IS in the dose of 200 mg/kg b.w./day; bpm, heart beats per minutes; IS, indoxyl sulfate; NS, non-significant; SpO_2_, blood oxygen saturation*.

### Relationships Between Parameters of Hemostasis and Concentrations of IS

Among the analyzed parameters of the hemostatic system, only TF/VII complex, FXa, and PAI-1 were associated positively with concentrations of IS. In turn, FV correlated inversely with concentrations of IS in plasma (Table 6).

**Table 6:** Relationships between parameters of hemostasis and concentrations of IS.

	Correlation coefficient, R	*p*-Value
PF4	0.2262	NS
vWF	0.0365	NS
TF	-0.0507	NS
TF/VII complex	**0.3441**	**0.0320**
FV	-**0.3798**	**0.0171**
FXa	**0.3516**	**0.0414**
TAFI	-0.2214	NS
PAI-1	**0.3355**	**0.0455**
PAP	-0.2383	NS
D-dimers	-0.2563	NS

*FV, coagulation factor V; FXa, activated coagulation factor X; NS, non-significant; PAI-1, plasminogen activator inhibitor-1; PAP, plasmin-alpha2-antiplasmin complex; PF4, platelet factor 4; TAFI, thrombin activatable fibrinolysis inhibitor; TF, tissue factor; TF/VII complex, complex of tissue factor and coagulation factor VII, vWF, von Willebrand factor. Bold values mean that correlation was statistically significant*.

## Discussion

The present study is a continuation of our previous work (Karbowska et al., 2017) and it also confirms prothrombotic properties of IS. We demonstrated that chronic exposure to IS promotes arterial thrombosis, increases kinetics and strength of clot formation, and enhances activity of platelet. In addition, in this work, we show for the first time that the prothrombotic state created by IS is associated with disturbances in hemostatic parameters, particularly, increased levels of complex TF/VII and PAI-1 and decreased level of PAP. Furthermore, we found that IS reduces SIRT1 and SIRT3 aortic contents.

To investigate the prothrombotic properties of IS, two doses of the mentioned toxin (100 and 200 mg/kg of b.w.) were administered chronically to rats. The doses used were enough to obtain significant elevations of IS in rats’ plasma and reflect level of IS observed in patients with CKD (Kaminski et al., 2017). A definite advantage of our model is a possibility to observe primarily an impact of IS without greater interferences from other toxins creating uremic milieu, which is impossible in models of CKD. It is well-recognized that IS exhibits nephrotoxicity due to its pro-oxidant and pro-inflammatory activities, and thus, it is also engaged in the vicious cycle of progressive CKD (Karbowska et al., 2016; Fujii et al., 2018). The examination of biochemical parameters in our study showed modest increase in the levels of plasma creatinine and UA and moderate augmentation of urine protein excretion. A slight reduction of creatinine clearance was also observed. Nevertheless, other renal function parameters like urea, BUN, plasma total protein, and urine creatinine did not differ. Moreover, a histological evaluation of kidneys did not reveal any evident alterations in kidney structure. Thus, despite observed biochemical changes, we hypothesize that renal compensatory mechanisms are still efficient. Differences in the concentration of IS in urine between used IS doses can provide evidence that kidney function was still effective. Therefore, it seems that our model allows to observe primarily the effect of IS without greater accumulation of other uremic toxins caused by kidney damage.

Our previous study demonstrated an acceleration of thrombotic response following vascular injury after acute exposure to IS (Karbowska et al., 2017). Similar results were obtained after chronic exposure to IS in the current work. We found that IS administered for 4 weeks led to an increased incidence of thrombosis as well as augmented thrombus weight, which were not accompanied by any alterations in cardiorespiratory parameters. Our data are also in line with the study of Yang et al. (2017), who used the carotid artery thrombosis model in mice. Further, they linked IS-induced thrombosis with platelet hyperactivity. An analysis of morphological parameters and ELISA kits from our study indicated that chronic exposure to IS not only results in an increased number of PLT, but also enhances their activity that was reflected by an increase in the level of PF4. The mentioned observations are in compliance with data presented in our previous work, which showed that IS-induced platelet accumulation in the area of endothelium laser injury resulted in augmented total thrombus area (Schroeder et al., 2010; Karbowska et al., 2017). Moreover, IS is a well-known AhR agonist. Some recent studies provide evidence that AhR may be involved in megakaryocyte differentiation and polyploidization. Lindsey and Papoutsakis (2011) showed that AhR-knockout mice had lower platelet counts. This may explain an increased PLT observed in our study. In addition, Lindsey et al. (2014) indicates AhR as an important element of platelet response to collagen (Pombo et al., 2015). The above-mentioned fact can elucidate the observation from our previous (Karbowska et al., 2017) and other works (Yang et al., 2017) regarding IS-induced exacerbation of collagen-induced aggregation of platelet in whole blood. Altogether, IS as an AhR agonist can increase the number of PLT, enhance their activity, and contribute to potentiation and priming of platelets activation by collagen. As a result, these effects may lead to augmented formation of platelet plug and, thereby, can result in increased thrombus weight, which is observed in our study.

In addition to the earlier observations, a histological evaluation revealed that thrombi of IS-treated rats were richer in fibrin with PLT when compared with control rats. This fact suggests that IS not only contributes to an increase in PLT, but also to increases the amount of fibrin. The above-mentioned hypothesis is also supported by data collected from our previous study, which showed that IS significantly enhances fibrin generation (Karbowska et al., 2017). Moreover, an analysis of the coagulation profile after chronic exposure to our toxin indicated that IS augments the concentration of fibrinogen — the precursor of fibrin. However, fibrinogen is an acute-phase protein as well. Therefore, it is possible that the increase in fibrinogen concentration observed is associated with inflammatory response as we also noticed augmentation in WBC.

Generally, the platelet plug formed during primary hemostasis is not stable. In turn, ROTEM analysis showed that IS induces an increase in MCF, a parameter reflecting the strength of the clot formed. Augmented stability of clots after IS administration can also be one of the causes of increased thrombosis incidence, which is observed in our model. Therefore, we hypothesize that IS not only enhances primary hemostasis, but also affects secondary hemostasis through influence on plasma coagulation and fibrinolysis factors. In addition, an IS-induced increase in clot firmness seems to be linked to slight impairment of fibrinolysis that was reflected by a decreased level of PAP and increased concentration of PAI-1. Moreover, the level of PAI-1 positively correlated with the concentration of IS. Obtained data are in line with work of Motojima et al., in which they observed that IS is able to upregulate gene expression of PAI-1 in human renal proximal tubular cells (Motojima et al., 2003). A further, increase in MCF is correlated with changes in fibrinolytic parameters. We have also noticed that IS contributes to a significant decrease in time from the start of the measurement to stable clot formation — CFT. The shortening of CT was not statistically significant; that is, it was in line with the obtained coagulation parameters. However, we consider that reduced CFT is associated with IS-induced changes in coagulation parameters, especially with an increased level of complex TF/VII, which was also positively correlated with the IS level. The tissue factor, as a receptor for factor VIIa, is a main initiator of blood coagulation. There are a lot of literature data available regarding influence of IS on TF. In particular, IS contributes to augmented stability and activity of TF via the AhR pathway, and these activities are definitely components of IS prothrombotic properties (Chitalia et al., 2013; Sallée et al., 2014; Ito et al., 2016; Shivanna et al., 2016; Kolachalama et al., 2018). Alongside an augmented concentration of complex TF/VII, we have also observed an IS-induced increase in the level of FXa, which was correlated with the concentration of IS and a decrease in the level of FV. A reduced level of FV can be the result of shifting balance to the activated form of FV or can be connected with an increased number of PLT, in which it is stored. Patients with CKD exhibit a hypercoagulable state, which can be represented by elevated concentrations of hemostatic parameters like TF/VII, PAI-1, and fibrinogen (Mercier et al., 2001; Segarra et al., 2001; Gondouin et al., 2013; Shashar et al., 2015). The results of our study are similar to these. However, to confirm that all of the observed changes in hemostatic parameters are not caused by IS-induced liver disorders (as we observed an increase in ALAT concentration), we performed histological evaluations of livers. Obtained data did not reveal any differences between control and IS-treated groups.

Recently, particular attention is being paid to Sir2 proteins — sirtuins. Hitherto, the role of sirtuins described is mainly associated with protection against senescence, oxidative stress, or inflammation (Winnik et al., 2015). However, some studies also show that sirtuins, especially Sirt1 and Sirt3, are involved in thromboprotection. The authors not only correlated the loss of sirtuins with the promotion of arterial thrombosis, but also demonstrated that their inhibition is linked to an elevated level of TF (Breitenstein et al., 2011; Gaul et al., 2018). In addition, Gaul et al. (2018) reported that arterial thrombosis in mice with Sirt3 knockout is enhanced with an increased formation of NETs. Little is known about the influence of IS on Sirt1 and Sirt3 and its involvement in CKD thrombosis. Koizumi et al. (2014) showed an IS-induced reduction in Sirt1 activity in human umbilical endothelial cells culture and tied it to AhR activation. Furthermore, Jeong et al. (2016) demonstrated that uremic toxins, including IS, induce *in vitro* formation of the DNA-histone complex, which is a marker for the formation of NETs. In the current work, we show for the first time that chronic exposure to IS leads to reduced aortic contents of Sirt1 and Sirt3 in rats, which, in turn, can contribute to a prothrombotic state. Thus, enhancing the activity of Sirt1 and Sirt3 may provide new therapeutic options for the treatment of thrombosis in patients with CKD with an elevated level of IS. However, data obtained in our study showed that both IS doses reduced the level of SIRT1 and SIRT3 at a similar magnitude, whereas changes in hemostatic parameters differed depending on the IS dose. Therefore, IS-mediated changes in Sirt1 and Sirt3 definitely need to be assessed at transcriptional and post-transcriptional levels, and the molecular mediators of these regulations should be determined to better understand the contribution of Sirt to the thrombotic process. In addition, due to the fact that uremic milieu consists of toxins other than IS, further studies are required to confirm changes in sirtuins in well-established models of CKD.

## Conclusion

This study demonstrates that IS promotes arterial thrombosis and prothrombotic state that is connected with an increased level of complex TF/VII, PAI-1, and platelet activation, as well as decreased aortic contents of SIRT1 and SIRT3. Obtained data suggest that IS enhances primary hemostasis leading to an augmented formation of platelet plug with an increased amount of fibrin and affects secondary hemostasis through influence of plasma coagulation and fibrinolysis factors that results in increased kinetics and strength of clot formation. Our findings may help to better understand mechanisms leading to increased thrombotic events in patients with CKD with elevated level of IS. However, the model from the current work described solely the IS impact. Considering the above, further studies are necessary in order to reveal the influence of complex interactions between other uremic toxins on thrombotic events in patients with CKD.

## Author Contributions

MK, TK, and DP conceived and designed the experiments and wrote the paper. MK, TK, BZ, TD, TM, TR, AP, KP, and DP performed the experiments. MK, TK, TM, AP, KG-U, KP, and DP analyzed the data. All authors read and approved the final manuscript.

## Conflict of Interest Statement

The authors declare that the research was conducted in the absence of any commercial or financial relationships that could be construed as a potential conflict of interest.

## Abbreviations

100 IS: group receiving indoxyl sulfate in the dose of 100 mg/kg b.w./day
200 IS: group receiving indoxyl sulfate in the dose of 200 mg/kg b.w./day
AhRs: aryl hydrocarbon receptors
ALAT: alanine aminotransferase
APTT: activated partial thromboplastin time
ASAT: aspartate aminotransferase
AT III: antithrombin III
AUC: area under the curve
b.w.: body weight
BUN: blood urea nitrogen
CFT: clot formation time
CKD: chronic kidney disease
CT: clotting time
CVDs: cardiovascular diseases
FV: coagulation factor V
FXa: activated coagulation factor X
H&E: hematoxylin and eosin
HCT: hematocrit
HDL-d: high-density lipoprotein direct
HGB: hemoglobin
HPLC: high-performance liquid chromatography
IS: indoxyl sulfate
LDL-d: low-density lipoprotein direct
MCF: maximum clot firmness
MCH: mean corpuscular hemoglobin
MCHC: mean corpuscular hemoglobin concentration
MCV: mean corpuscular volume
NETs: neutrophil extracellular traps
PAI-1: plasminogen activator inhibitor-1
PAP: plasmin-alpha2-antiplasmin complex
PAS: periodic acid schiff
PF4: platelet factor 4
PLT: platelet
PT: prothrombin time
RBCs: red blood cells
Sir2: silent information regulator 2
Sirt1: sirtuin 1
SIRT1: sirtuin 1
Sirt3: sirtuin 3
SIRT3: sirtuin 3
SpO_2_: blood oxygen saturation
STUB1: STIP1 homology and U-box containing protein 1
TAFI: thrombin activatable fibrinolysis inhibitor
TF: tissue factor
TF/VII complex: tissue factor/coagulation factor VII complex
TGs: triglycerides
TT: thrombin time
UA: uric acid
vWF: von Willebrand factor
WBC: white blood cells.
